# Crystal Structure and Activity Analysis of *Chlamydophila pneumoniae* AP Endonuclease IV

**DOI:** 10.3390/biom16040594

**Published:** 2026-04-17

**Authors:** Jinglin Jin, Yitong Zhang, Shiyang Guo, Lihong Yang, Haixia Liu, Long Liu, Wei Gao

**Affiliations:** School of Science, Beijing Forestry University, 35 Qinghuadong Road, Beijing 100083, China; jinglin04@bjfu.edu.cn (J.J.); zytong42@bjfu.edu.cn (Y.Z.); guoshiyang@bjfu.edu.cn (S.G.); lihongyang@bjfu.edu.cn (L.Y.); liuhx@bjfu.edu.cn (H.L.); xiyangyang@bjfu.edu.cn (L.L.)

**Keywords:** *Chlamydophila pneumoniae*, endonuclease IV, CpEndoIV, crystal structure, AP site, DNA repair

## Abstract

DNA damage requires repair via the endonuclease IV-mediated base excision repair (BER) pathway, which corrects apurinic/apyrimidinic (AP) sites. *Chlamydophila pneumoniae* AP endonuclease IV (CpEndoIV), the sole AP endonuclease in this pathogen, is crucial for genomic integrity. As humans lack a homologous protein, it represents a potential therapeutic target. In this study, we report the first crystal structure of CpEndoIV at 1.97 Å resolution. The structure reveals two Zn^2+^, one Mg^2+^, and a malonate molecule bound in the active site, marking the first observation of Mg^2+^ coordination in the EndoIV family. Compared to the three-Zn^2+^ model with a narrow, deep pocket for precise AP-site cleavage, the Zn^2+^/Mg^2+^-bound state has a wider, shallower pocket that might promote diverse catalytic activities. Combined with enzymatic assays, we suggest that the mixed Zn^2+^/Mg^2+^ model is better adapted for CpEndoIV to operate under host oxidative stress. Malonate binds to the metal ions, occupying the positions normally coordinated by water molecules. This binding mode may mimic the coordination of the substrate to the metal ions, and the protein conformation resembles that of the enzyme upon substrate binding at the active site. This study provides a structural basis for the functional characterization of CpEndoIV and offers a reference for the development of targeted inhibitors against diseases caused by *Chlamydophila pneumoniae*.

## 1. Introduction

DNA damage occurs in all living organisms, potentially arising from replication errors, toxins, ultraviolet (UV) radiation, and reactive oxygen species (ROS) [1]. This can exert potentially mutagenic and toxic effects on cells. Consequently, all organisms require DNA repair mechanisms to ensure faithful DNA replication [2]. DNA damage primarily encompasses cytosine deamination by hydrolysis, base loss, base oxidation, base methylation, base crosslinking, DNA strand breaks, and misincorporation during DNA replication [2,3,4]. Among these types of lesions, the most common damage is base loss, resulting in sites known as apurinic/apyrimidinic (AP) sites [5,6,7]. In mammalian cells, between 2000 and 10,000 AP sites are generated per cell per day due to spontaneous depurination/depyrimidination [8]. AP sites primarily originate from spontaneous base loss via depurination/depyrimidination, as well as from the removal of damaged bases by various DNA glycosylases [9]. Since AP sites lack a paired base, they cannot form the necessary hydrogen bonds to guide correct deoxynucleotide monophosphate (dNMP) incorporation, thereby potentially inhibiting replication or transcription.

In cells, apurinic/apyrimidinic (AP) sites are primarily repaired through the base excision repair (BER) pathway. BER is initiated by DNA glycosylases, which directly generate AP sites [9,10]. The enzymes responsible for cleaving the DNA backbone at AP sites during BER fall into two classes: AP lyases and AP endonucleases. AP lyases cleave the DNA backbone by a β-elimination reaction at the 3′ side of the AP site, generating a 5′-phosphate and a 3′-α,β-unsaturated aldehyde [11]. AP endonucleases cleave the DNA backbone through hydrolysis of the phosphodiester bond on the 5′ side of the AP site, producing a free 3′-OH group and a 5′-deoxyribose phosphate (dRP) moiety [12]. AP endonucleases primarily comprise two families: the bacterial endonuclease IV (Nfo) family and the exonuclease III (Xth) family. Nfo and its homologs are predominantly found in lower organisms, whereas Xth and its homologous proteins are ubiquitous in higher organisms [13].

Studies have demonstrated that both families possess not only AP endonuclease activity but also 3′-5′ exonuclease and 3′-phosphodiesterase activities [14,15]. Within AP endonucleases, the primary responsibility for 3′-5′ exonuclease activity lies with the exonuclease III family of homologous proteins, which function in a Mg^2+^-dependent manner [16,17]. While *Escherichia coli* EndoIV exhibits very weak 3′ exonuclease activity, this activity is stronger in some species that possess only EndoIV [18]. However, the mechanistic basis for the 3′ exonuclease activity of EndoIV remains poorly understood. A previously solved structure of an H69A mutant EndoIV in complex with a duplex DNA containing an α-anomeric 2’-deoxyadenosine:T pair revealed that the aberrant 3′-terminal dC is positioned near the cleavage site in the enzyme’s active site [19]. However, the detailed mechanism of cleavage has not been further explored.

The AP endonuclease activity of EndoIV involves an evolving understanding of its catalytic mechanism. Early studies proposed a three-zinc model, in which two Zn^2+^ bridge a water molecule to generate a nucleophile that attacks the phosphodiester bond phosphorus, leading to cleavage [13]. Later research suggested that Glu261 (in EcoNfo), coordinated to one Zn^2+^, deprotonates a water molecule to create the nucleophile for hydrolyzing the phosphodiester bond [16,20]. Most recently, it was found that in EndoIV from Gram-positive bacteria, a Tyr33 replaces the corresponding Glu261 and, together with the metal ion originally bound by Glu261, coordinates a water molecule [21].

The currently resolved structures of EndoIV from various species can be broadly categorized based on their metal ion cofactors: the three-zinc model, the zinc-manganese mixed model, the zinc-iron mixed model, and the zinc-calcium mixed model [12,16,20,21,22,23,24,25]. Representative structures of the three-zinc model include those from *Escherichia coli* (PDB id 1QTW), Bacillus anthracis (PDB id 1XP3), *Mycobacterium tuberculosis* (PDB id 5ZHZ), *Thermotoga maritima* (PDB id 2X7V), and the African swine fever virus (PDB id 6KI3). The zinc-manganese and zinc-calcium models have, to date, only been reported in *Thermotoga maritima* (PDB id 4HNO, 2X7W). The zinc-iron model has been reported in *Geobacillus stearothermophilus* (PDB id 3AAL) and Staphylococcus aureus (PDB id 8AXY). Multiple enzymatic activity studies on EndoIV enzymes indicate that different species exhibit distinct preferences for metal ions [4,19,20,21,26]; however, the mechanistic basis for this selectivity remains unclear.

*Chlamydophila pneumoniae* (*C. pneumoniae*) is a Gram-negative, obligate intracellular pathogen classified as a bacterium based on its cell wall composition and growth by binary fission, exhibiting a unique biphasic developmental cycle. Its genome is exceptionally small, encoding only 1116 open reading frames (ORFs). Infection begins when *C. pneumoniae*, in its small, infectious elementary body (EB) form, attaches to and is phagocytosed by respiratory epithelial cells. Inside a host-derived vacuole (the inclusion), it differentiates into the larger, metabolically active reticulate body (RB) form for replication [27,28]. This pathogen has been implicated in respiratory infections and pneumonia, as well as in several chronic conditions such as Alzheimer’s disease, atherosclerosis, and multiple sclerosis [29,30,31]. Under stress conditions, *C. pneumoniae* can persist long-term within host cells, leading to chronic inflammation and tissue damage [32,33]. It is also involved in the pathogenesis of atherosclerosis by inducing oxidative stress [32], indicating that *C. pneumoniae* itself resides in an oxidative stress environment. The *Chlamydophila pneumoniae* AP endonuclease IV (CpEndoIV) is the sole AP endonuclease in this organism [34]. It exhibits not only AP endonuclease activity [35] and 3′-5′ exonuclease activity that can substitute for DNA polymerase I [23], but also 3′-phosphodiesterase activity [36]. However, structural information for CpEndoIV remains unavailable. Furthermore, its reported promiscuity for various metal ions and its broader functional repertoire compared to homologs have resulted in limited mechanistic insights into how CpEndoIV catalyzes its multiple activities.

Therefore, in this study, we have determined the structure of *Chlamydophila pneumoniae* AP endonuclease IV for the first time, identifying two Zn^2+^ and one Mg^2+^ bound at its active site. These three metal ions are simultaneously coordinated by a malonate molecule. Comparative structural analysis with homologs, considering its AP endonuclease and 3′-5′ exonuclease activities, led us to propose that the mixed Zn^2+^/Mg^2+^ binding model is more conducive for *Chlamydophila pneumoniae* AP endonuclease IV to perform its diverse cleavage activities compared to the three-Zn^2+^ model. Furthermore, this structural study reveals that malonate chelates the metal ions by occupying the coordination sites typically filled by water molecules. This binding mode likely mimics the coordination of the substrate to the metal ions. This work provides a structural basis for understanding CpEndoIV and offers a reference for the structure-based design of targeted inhibitors against diseases caused by Chlamydia pneumoniae, such as chlamydial pneumonia and asthma.

## 2. Materials and Methods

### 2.1. Strains and Reagents

The full-length gene was kindly provided by the research group of Professor Xipeng Liu from Shanghai Jiao Tong University. The expression vector pET28a and the *Escherichia coli* strain BL21(DE3) were preserved in our laboratory. Primers were synthesized by Genewiz (Suzhou, China). Restriction endonucleases NdeI and XhoI, DNA polymerase, T4 DNA ligase, DNA markers, and protein markers were purchased from TaKaRa Bio (Dalian, China). DNA gel extraction and plasmid extraction kits were purchased from Tiangen Biotech (Beijing, China). Isopropyl β-D-1-thiogalactopyranoside (IPTG), phenylmethylsulfonyl fluoride (PMSF), and kanamycin were purchased from Merck KGaA (Darmstadt, Germany). Nickel–nitrilotriacetic acid (Ni–NTA) affinity chromatography columns and Superdex 200 size-exclusion chromatography columns were purchased from GE Healthcare Life Sciences (Chicago, IL, USA). Crystallization screening kits, including PEG/Ion 2 Screen, were purchased from Hampton Research (Aliso Viejo, CA, USA).

### 2.2. Cloning, Expression and Purification

**Figure 1:**
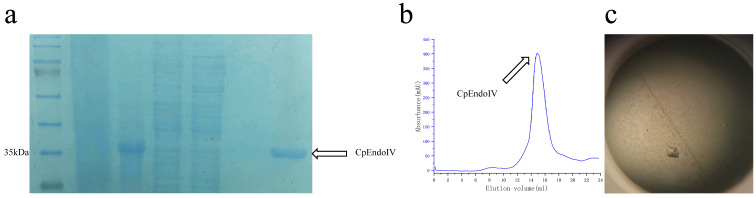
Expression, purification and crystallization of CpendoIV. (**a**) SDS-PAGE of CpendoIV expressed in *E. coli* BL21 (DE3) cells. The lanes represent the samples before induction, after induction, and after Ni-NTA affinity purification, with the target protein migrating at approximately 35 kDa. (**b**) Purification of CpEndoIV using 900 mM NaCl lysis buffer yielded a single symmetric peak on a Superdex G200 SEC column. (**c**) A crystal of CpEndoIV.

The full-length CpEndoIV gene from *Chlamydophila pneumoniae* was amplified by polymerase chain reaction (PCR). The PCR product and the pET-28a (+) plasmid were digested with restriction enzymes and ligated using T4 DNA ligase. Successful cloning was confirmed by Sanger sequencing. The verified recombinant plasmid was then transformed into *Escherichia coli* BL21(DE3) competent cells. A single colony was selected and cultured in Luria–Bertani (LB) medium supplemented with 50 µg/mL kanamycin. The culture was grown at 37 °C until the optical density reached approximately 0.6–0.8. Protein expression was induced by adding 0.4 mmol/L isopropyl β-D-1-thiogalactopyranoside (IPTG), followed by incubation at 16 °C for 18 h. The induced cells were harvested by centrifugation at 3500× *g* for 35 min at 4 °C. The cell pellet was resuspended in lysis buffer containing 900 mmol/L NaCl, 10% (*v*/*v*) glycerol, 1 mmol/L phenylmethylsulfonyl fluoride (PMSF), and 20 mmol/L Tris-HCl (pH 7.8), and then lysed by ultrasonication. The soluble fraction was separated by centrifugation and subjected to nickel–nitrilotriacetic acid (Ni–NTA) affinity chromatography. The His-tagged CpEndoIV was eluted using an imidazole gradient. Protein purity and concentration were analyzed by SDS-PAGE and the Bradford method (Figure 1a). Subsequently, high-purity monomeric CpEndoIV was obtained by size-exclusion chromatography (Figure 1b). Other detailed experimental procedures are described in our previous article [37].

### 2.3. Crystallization and Data Collection

Initial crystallization conditions for His-tagged CpEndoIV were screened via the sitting-drop vapor diffusion method, and the crystallization conditions were subsequently optimized using the hanging-drop vapor diffusion method. Crystals were grown at 16 °C by equilibrating a mixture of 1 μL reservoir solution (8% *v/v* Tacsimate, pH 5.2, 25% *w/v* PEG 3350) and 1 μL protein solution (2.81 mg/mL protein in SEC buffer: 100 mmol/L NaCl, 20 mmol/L Tris-HCl, pH 7.8) against 400 μL reservoir solution in a crystallization plate. Crystals appeared after 7 days of incubation (Figure 1c). X-ray diffraction data were collected at beamline BL02U1 of the Shanghai Synchrotron Radiation Facility (SSRF). During data collection, the crystals were cryoprotected using a solution consisting of the reservoir solution (8% *v/v* Tacsimate, pH 5.2, 25% *w/v* PEG 3350) supplemented with 20% (*v*/*v*) glycerol. The diffraction data were processed and scaled using the HKL2000 program. Meanwhile, the remaining crystals from the same batch were subjected to mass spectrometry analysis to determine the types and relative abundance of metal ions present within the crystals.

### 2.4. Structure Determination, Refinement and Analysis

The Matthews coefficient and solvent content were calculated using the cell content analysis program in the CCP4 suite and were cross-validated using the Xtriage program in Phenix. Initial phases were obtained by molecular replacement using Phaser MR, with an AlphaFold2-predicted model serving as the search model. The resulting structure was then subjected to multiple rounds of iterative refinement using Coot and Phenix refine, yielding the final three-dimensional structure of CpEndoIV. Details of the crystallographic data collection and refinement statistics are provided in Table 1. Structural analysis and figure preparation were performed using ChimeraX 1.9 [38]. Amino acid sequence alignment analysis was performed using ESPript 3.2 (https://espript.ibcp.fr/ESPript/ESPript/index.php, accessed on 14 January 2026).

## 3. Results and Discussion

### 3.1. Overall Structure of CpEndoIV

**Figure 2:**
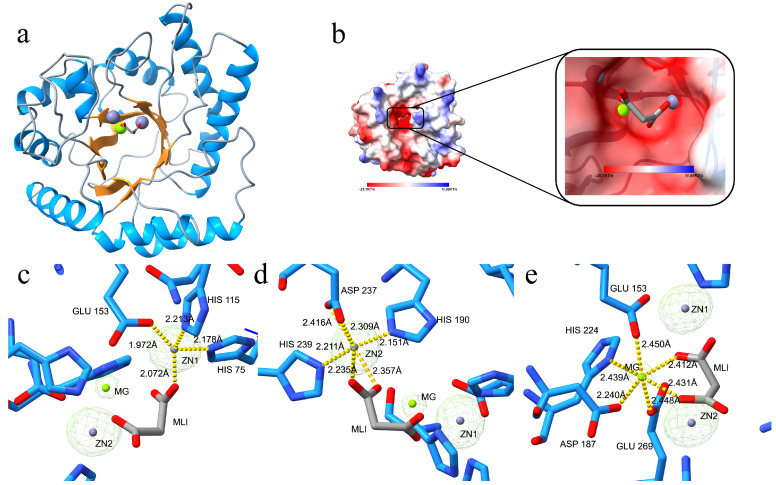
Overall structure of CpEndoIV and coordination environment of the metal ions with the F_obs_ − F_calc_ difference map (green) corresponding to the metal coordination. (**a**) The three-dimensional structure of CpEndoIV is shown colored by secondary structure, with α-helices in dodger blue, β-strands in orange, and loops in off-white. Zn^2+^ is represented as a gray sphere, Mg^2+^ as a green sphere, and the malonate molecule is shown as gray sticks. (**b**) Electrostatic surface potential map of CpEndoIV and a close-up view of its active site, showing the solvent exposure of Mg^2+^ and one Zn^2+^. (**c**) The green density represents the F_obs_ − F_calc_ difference map calculated before the incorporation of ZN1, ZN2 and MG, contoured at 7σ. The peaks corresponding to ZN1 and ZN2 were each at 35σ, while the MG site showed a peak height of 12σ. ZN1 coordinates with the N atoms of His75 and His115, an O atom of Glu153, and an O atom of malonate (MLI) in a tetrahedral geometry, with bond lengths indicated. (**d**) ZN2 is coordinated by the N atoms of His190 and His239, O atoms of Asp237 and Asp187, and bidentate coordination with two O atoms of malonate, forming an octahedral geometry, with bond lengths displayed. (**e**) MG forms an octahedral coordination sphere with the N atom of His224, O atoms of Glu153, Glu269, and Asp187, and one carboxylate O atom from each of the two carboxylic groups of malonate, with corresponding bond lengths shown.

The final model of CpEndoIV was refined to a resolution of 1.97 Å, with Rwork and Rfree values of 0.17 and 0.20, respectively (Table 1). The CpEndoIV structure comprises 287 amino acids in its full-length sequence. Due to high flexibility in the N-terminal region, the first two residues lacked clear electron density, and therefore the model begins from the third residue, VAL. One asymmetric unit contains two identical protein molecules, with a root-mean-square deviation (RMSD) of 0.131 Å for 260 aligned Cα atoms. CpEndoIV is a single-domain protein, featuring eight parallel β-strands (β1 to β8) surrounded by eight peripheral α-helices (α1 to α8), forming a TIM barrel fold (Figure 2a). This α/β-barrel fold was initially discovered in triose phosphate isomerase (TIM) and defines a major superfamily found in numerous enzymes with diverse functions [39,40]. In CpEndoIV, the N-termini of all eight flanking α-helices point toward the C-terminal end of the β-barrel. A protruding loop at the C-terminal end of the β-barrel connects the α-helices and β-strands, creating a deep groove. Three metal ions and the majority of conserved amino acids are located at the bottom of this groove, indicating that this region constitutes the active site pocket. According to complex structures of homologous proteins, interaction with double-stranded DNA is mediated by the DNA recognition loops (R-loops) at the C-terminal end of the central β-barrel. These R-loops participate in AP-DNA substrate binding by engaging the phosphate backbone and providing base interactions at the AP site [12].

To identify the metal ion species in the active site, ICP-MS analysis was performed on dissolved protein crystals. The results revealed abundant Na^+^, modest amounts of Zn^2+^, Mg^2+^, Mn^2+^, and trace Ni^2+^ in the solution. During structure refinement, prominent F_obs_ − F_calc_ difference electron density peaks were observed at the active site in Coot (Figure 2c). The signals remained strong when contoured at 7σ, showing intensities well above the background level with well-defined density distribution. The F_obs_ − F_calc_ difference map showed a peak height of 12σ at the third site, which was substantially lower than the peak heights at the two zinc sites (35σ). This observation indicated that zinc, manganese, or other transition divalent metal ions with comparable scattering power are unlikely to bind at this site with full or near-full occupancy, whereas a light divalent metal such as magnesium is feasible. Upon the addition of magnesium, the positive green peak in the F_obs_ − F_calc_ difference map disappeared, no abnormal B-factors were observed, and the R-factors decreased significantly. In contrast, upon addition of zinc or manganese, strong negative (red) density appeared at this site. Combined with evidence from expression, purification, crystallization, mass spectrometry, and iterative refinement cycles, each metal ion (two Zn^2+^ and one Mg^2+^) was confirmed to be fully occupied, with refined occupancies of 1 and reasonable coordination geometries. Collectively, the active site of CpEndoIV was determined to contain two Zn^2+^ and one Mg^2+^, coordinated by His75, His115, Glu153, His190, His239, Asp237, Asp187, His229, Glu269, and a malonate molecule (Figure 2c–e).

### 3.2. Activity Analysis of CpEndoIV

#### 3.2.1. Role of Metal Ions in the AP Endonuclease and 3′-5′ Exonuclease Activities of CpEndoIV

The active site of CpEndoIV binds two Zn^2+^ and one Mg^2+^. The Mg^2+^ occupies a position typically coordinated by Zn^2+^ in other EndoIV enzymes, ligated by a set of highly conserved surrounding amino acid residues (Figure 2e). This represents the first observation of Mg^2+^ acting as a coordinating metal ion within the EndoIV family of proteins.

Consistent with previous enzymatic assays of CpEndoIV, which showed that the addition of Mg^2+^ or Zn^2+^ significantly enhanced its AP endonuclease activity and that activity was not completely abolished in the presence of EDTA [35], our structural determination now provides an explanation. The solved structure confirms that both Mg^2+^ and Zn^2+^ can indeed serve as coordinating metal ions for CpEndoIV to execute its AP endonuclease function.

The electron density map indicates that the peak intensity for Mg^2+^ is weaker compared to those for the two Zn^2+^ (Figure 2c), with longer coordination bond lengths to surrounding atoms (Figure 2e). Furthermore, the Mg^2+^ site is more solvent-exposed (Figure 2b). These observations suggest that the binding affinity of Mg^2+^ at this site is weaker and its dissociation rate faster than those of the two Zn^2+^. Consequently, we propose that, in the presence of EDTA, in addition to chelating free metals, EDTA preferentially targets this Mg^2+^ site to inhibit CpEndoIV activity. Although the binding affinity of EDTA for metals is weaker than that of the enzyme, the chelation between Mg^2+^ and EDTA is reversible. This reversibility likely leads to the establishment of an equilibrium state, resulting in the observed plateau in activity inhibition.

**Figure 3:**
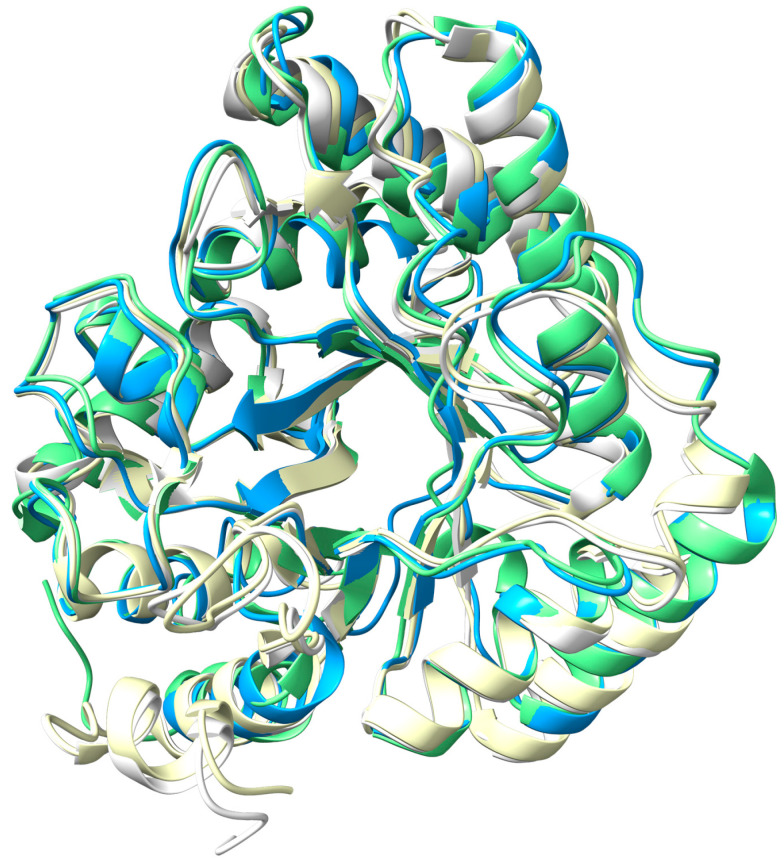
Structural comparison of CpEndoIV with homologous proteins from Gram-positive and Gram-negative bacteria. Structural comparisons are shown using *Escherichia coli* (1QTW, pale green) and *Chlamydophila pneumoniae* (dodger blue) as representatives of Gram-negative bacteria and *Geobacillus kaustophilus* (3AAL, white) and *Staphylococcus aureus* (8AXY, pale yellow) as representatives of Gram-positive bacteria, reflecting the structural differences that distinguish these two groups.

Structural alignment with homologous proteins (Figure 3 and Table 2) reveals a high degree of conservation among EndoIV-type proteins, with CpEndoIV showing the greatest similarity to the *E. coli* structure. Previous studies have demonstrated that the active site of EndoIV proteins can bind three Zn^2+^ to mediate cleavage [12]. Given that Zn^2+^ effectively enhances the AP endonuclease activity of CpEndoIV, with an efficiency comparable to that of Mg^2+^ [35], it is plausible that a three-Zn^2+^ binding model is also feasible for CpEndoIV, despite the absence of a structural model with three bound Zn^2+^.

These findings indicate that CpEndoIV exhibits considerable flexibility in metal ion selection for its AP endonuclease activity, rather than relying on a strictly specific, single-metal-dependent cleavage mechanism. This flexibility may also be attributed to the weaker binding affinity associated with the coordination geometry of the metal ion occupying this specific site.

Previous studies have shown that only the addition of Mg^2+^ significantly enhances the 3′-5′ exonuclease activity of CpEndoIV, whereas other metals such as Zn^2+^, Ca^2+^, and Mn^2+^ inhibit this activity [23]. This indicates that Mg^2+^ plays a crucial role in the 3′-5′ exonuclease function of CpEndoIV. Our structural data, revealing Mg^2+^ bound in the active site, provide direct evidence that CpEndoIV can incorporate Mg^2+^ as a cofactor for its 3′-5′ exonuclease activity. Furthermore, this suggests that the presence of Mg^2+^, compared to Zn^2+^, is more favorable for CpEndoIV to perform its diverse cleavage activities. It is plausible that the 3′-5′ exonuclease activity in CpEndoIV operates via a Mg^2+^-dependent cleavage mechanism.

A previous study on Mycobacterium tuberculosis endonuclease IV showed increased AP endonuclease activity with Mg^2+^ and Ca^2+^ present, but did not investigate whether Mg^2+^ affects its 3′-5′ exonuclease activity [26]. The later solved structure of this protein found bound Zn^2+^, and did not study the function of Mg^2+^ further [41]. Similarly, adding Mg^2+^ enhances the AP endonuclease activity of *Thermococcus eurythermalis* AP EndoIV (TeuEndoIV) [4], but no structural information is available for TeuEndoIV. None of the EndoIV structures determined so far have shown bound Mg^2+^. Therefore, understanding how Mg^2+^ works as a coordinating metal in the cleavage process requires high-resolution complex structures for further explanation.

#### 3.2.2. Comparative Analysis of the Active Site in Homologous Proteins

**Figure 4:**
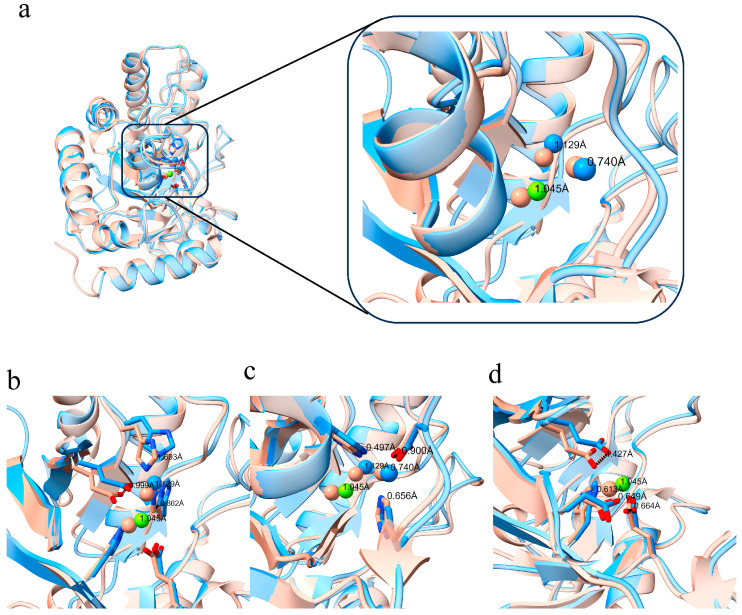
Superimposed structures of CpEndoIV (dodger blue) and *E. coli* EndoIV (dark salmon) and analysis of active-site geometry. (**a**) Overall structural superposition and detailed view of the active-site region. (**b**) Displacement of ZN1 and its coordinating residues. (**c**) Displacement of ZN2 and its coordinating residues. (**d**) Displacement of MG and its coordinating residues.

Structural alignment reveals that, compared to *E. coli* EndoIV, the positions of the three metal ions in CpEndoIV are shifted towards the bottom of the active site groove (Figure 4a). ZN1 is displaced outward by 1.129 Å, ZN2 by 0.74 Å, and MG by 1.045 Å, all within approximately 1 Å. Correspondingly, the coordinating amino acid residues for ZN1 move by a comparable distance of about 1 Å (Figure 4b). Residues coordinating ZN2 shift within a range of 0.5–0.9 Å (Figure 4c). For the residues coordinating MG, except for Asp, which shifts by 1.4 Å, the others move by around 0.6 Å, and their displacement directions are largely consistent (Figure 4d). Overall, the coordinated residues and metal ions move in a similar direction—towards the opening of the active site pocket—with displacements fluctuating around 1 Å. This results in a wider active pocket positioned closer to the barrel base in CpEndoIV, leading to greater solvent exposure of the metal ions, particularly Mg^2+^ (Figure 2b).

**Figure 5:**
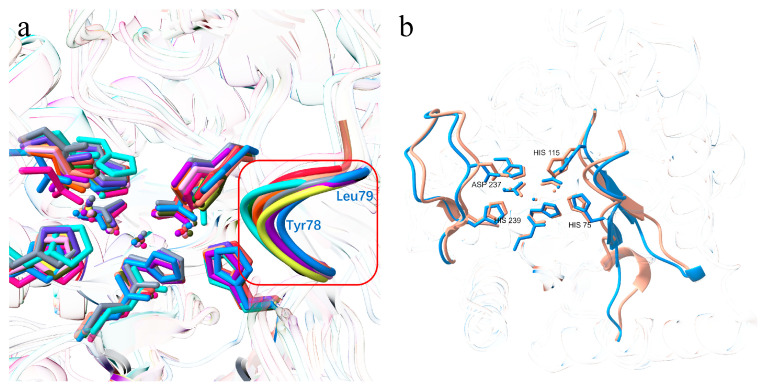
Structural superposition of the active-site entrance in CpEndoIV with EndoIV from *Escherichia coli* and other species. (**a**) *Chlamydophila pneumoniae* (dodger blue), *Escherichia coli* (monomer: 8RLY, dark violet; 2NQH, orange red; 1QTW, dark salmon; complex with bound DNA: 2NQJ, salmon; 2NQ9, hot pink; 1QUM, olive; 4K1G, green yellow), *Thermotoga maritima* (4HNO, slate blue; 2X7V, black; 2X7W, light slate gray), *Thermus thermophilus HB8* (3AAM, cyan (aqua)), *Geobacillus kaustophilus* (3AAL, dark green), *Staphylococcus aureus* (8AXY, Indian red), *Bacillus anthracis* (1XP3, red), *Mycobacterium tuberculosis* (5ZHZ, deep pink). The superposition highlights the structural alignment of Tyr78 and Leu79 (within the red box), as well as the displacement of active-site metal ions. (**b**) Close-up view comparing the loop regions near the active-site pocket between *E. coli* EndoIV (dark salmon) and *C. pneumoniae* EndoIV (dodger blue), showing pronounced shifts for Asp237, His239, His75, and His115.

Compared to other EndoIV enzymes, the metal ions in CpEndoIV are farther apart from each other and have longer coordination bond lengths with surrounding residues, which effectively expands the active site. Additionally, Tyr78 is shifted to the right relative to Tyr72, further contributing to a wider and larger pocket (Figure 5a). In contrast, the *E. coli* EndoIV pocket is deeper and narrower, with bond lengths within the optimal range for stable metal coordination. This suggests that in the three-Zn^2+^ bound state, the protein’s active site engages in rigid coordination with the Zn^2+^. The deeper, narrower, and more enclosed conformation is likely more conducive to the precise, specific cleavage of AP sites.

To address whether the larger active pocket in CpEndoIV is primarily due to the different metal ions or is an inherent structural adaptation for multiple cleavage activities, the structural superposition (Figure 5a) shows that even when coordinating different metals, the metal-binding residues in CpEndoIV still exhibit significant displacement. This indicates that, while metal coordination has an influence, the predominant reason is that the active site architecture of CpEndoIV itself is shifted more towards the base compared to other homologs.

In CpEndoIV, the α-helices are shorter, and the loop regions are longer, particularly in the DNA-binding loops. This results in greater displacements for key residues such as His239, Asp237, His75, and His115, which are predominantly located within these loops (Figure 5b). Previous studies have shown that CpEndoIV, as the sole AP endonuclease in C. pneumoniae, possesses activities for recognizing and cleaving diverse substrates. The shallower and wider pocket may facilitate easier substrate capture by the enzyme, and the longer coordination bonds may also favor substrate release. These features may represent advantageous evolutionary adaptations in CpEndoIV, enabling rapid substrate capture and efficient DNA damage repair within the host cell environment.

*Chlamydophila pneumoniae* primarily infects cells of the respiratory system, an environment rich in reactive oxygen species. Recent studies have linked *C. pneumoniae* to various chronic infections, noting its ability to induce host oxidative stress [32,33]. This, in turn, can trigger host nutritional immunity that restricts the availability of metal ions such as Fe^2+^/Fe^3+^, Zn^2+^, and Mn^2+^. However, due to its high abundance, Mg^2+^ concentration is difficult for the host to limit [42]. Therefore, within this same oxidative environment, *C. pneumoniae* may be compelled to rely more readily on Mg^2+^ for CpEndoIV to repair its genomic DNA damage—an evolutionary compromise for the pathogen. Consequently, we propose that a mixed Zn^2+^/Mg^2+^ binding model is likely better adapted for CpEndoIV to perform its diverse cleavage functions under oxidative stress conditions compared to a three-Zn^2+^ model.

### 3.3. Coordination Analysis of Malonate with CpEndoIV Suggests a Potential Inhibitory Effect

**Figure 6:**
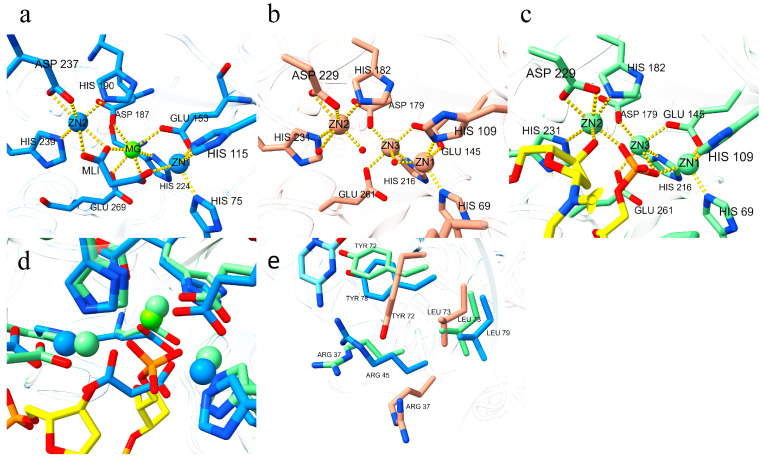
Active-site comparison of CpEndoIV (dodger blue) with the monomeric (dark salmon) and DNA-bound complex (light green) forms of *Escherichia coli* EndoIV. (**a**) In CpEndoIV, all remaining coordination sites on the metal ions (in addition to the protein residues) are occupied by the central malonate (MLI) molecule, forming a stable coordination architecture. (**b**) Coordination state in the *E. coli* EndoIV monomer, with red spheres representing water molecules. The remaining coordination sites on the three metal ions are all occupied by water, forming a stable complex. (**c**) Coordination state in the catalytic center of *E. coli* EndoIV bound to AP-DNA. The yellow structure represents the ribose with a 3′-OH group, and the orange structure denotes the phosphate group bound to the ribose. The remaining coordination sites on the zinc ions bind to the phosphate group and the 3′-OH, respectively. (**d**) Superposition of the catalytic regions between CpEndoIV and the *E. coli* complex. The two carboxylate groups of malonate overlap with the two oxygen atoms of the phosphate group and the position of the 3′-OH. (**e**) Structural alignment of CpEndoIV with the monomeric and complexed forms of *E. coli* EndoIV, focusing on residues Tyr78 (Tyr72), Leu79 (Leu73), and Arg45 (Arg37).

Our structure reveals that the two carboxylate oxygens of the malonate molecule coordinate to the two Zn^2+^, denoted as Zn1 and Zn2. Zn1 adopts a tightly bound tetrahedral geometry with four-coordinate ligation, while Zn2 forms a distorted octahedral geometry with six-coordinate chelation. Simultaneously, the malonate forms a secondary coordination interaction with Mg^2+^ at a distance of 2.4 Å, occupying the remaining two sites in the octahedral coordination sphere of Mg^2+^ (Figure 6a). This multidentate coordination occupies the sites on Zn^2+^ and Mg^2+^ that are normally available for binding water molecules (Figure 6b), potentially preventing the enzyme from activating the nucleophile and locking it in an inhibited state.

Furthermore, comparison with the complex structures of homologous enzymes (Figure 6c,d) shows that the coordination mode between malonate and the metal ions resembles that observed when CpEndoIV binds to an apurinic/apyrimidinic (AP) site in AP DNA. We also observed that the residues in CpEndoIV corresponding to Arg37, Tyr72, and Leu73 in other EndoIV enzymes—which insert into the DNA minor groove—are Arg45, Tyr78, and Leu79. In our structure, these residues adopt a conformation more similar to the substrate-bound state than to the apo state (Figure 6e). Our previous work has confirmed the involvement of these residues in DNA binding [37]. These findings suggest that the binding of malonate to CpEndoIV may, to some extent, mimic the coordination of the substrate with the metal ions. This potential inhibitory effect requires further validation through subsequent enzyme activity assays.

## 4. Conclusions

In summary, this study determined the high-resolution structure of CpEndoIV through cloning, expression, purification, and crystallization. The structure reveals that CpEndoIV binds one Mg^2+^ and two Zn^2+^, which are simultaneously coordinated by a malonate molecule. This represents the first observation of Mg^2+^ acting as a coordinating metal within the EndoIV protein family. Consistent with previous enzymatic assays, this finding directly implicates Mg^2+^ as a cofactor in the multiple catalytic activities of CpEndoIV. Comparative structural analysis indicates that EndoIV enzymes binding three Zn^2+^ possess a narrower and deeper active site pocket, conducive to the precise cleavage of AP sites. In contrast, the Zn^2+^/Mg^2+^-bound state exhibits a wider and shallower active pocket, which may facilitate a broader range of enzymatic activities. While the current structural evidence confirms that Mg^2+^ directly participates as a coordinating metal in both the endonuclease and exonuclease activities of CpEndoIV, elucidating the precise mechanistic role of Mg^2+^ in the cleavage process will require high-resolution structures of the enzyme in complex with various DNA substrates.

Furthermore, the structure shows that malonate binds to the coordinating metals by occupying the water-coordination sites on the metal ions and that the conformations of key amino acid residues in the DNA-binding region of the enzyme resemble those in the substrate-bound state, suggesting that malonate may have potential inhibitory activity. Future biochemical experiments, such as assessing the effects of malonate on substrate-binding affinity as well as AP endonuclease and exonuclease activities, are required to validate the function of malonate. This provides a valuable reference for the development of targeted therapies against CpEndoIV.

## Figures and Tables

**Table 1:** Data collection and structure refinement. Values for the outer shell are given in parentheses.

Data Collection	
Diffraction source	SSRF BL02U1
Wavelength (Å)	0.979183
Crystal-detector distance (mm)	228.000
Rotation range per image (°)	1.000
Total rotation range (°)	360
Exposure time per image (s)	0.100
Temperature (K)	100
Space group	*P*2_1_2_1_2_1_
a, b, c (Å)	67.93, 68.08, 150.84
α, β, γ (°)	90, 90, 90
Resolution range (Å)	62.05–1.97 (2.02–1.97)
Total No. of reflections	636,996 (37,499)
No. of unique reflections	50,308 (3653)
Completeness (%)	100.0 (100.0)
Redundancy	12.7 (10.3)
⟨I/σ(I)⟩	18.8 (2.0)
R_r_._i_._m_.	0.077 (1.316)
Wilson B-factor(Å^2^)	39.4
**Refinement**	
Resolution range (Å)	31.026–1.97
Completeness (%)	99.95 (100.00)
No. of reflections, working set	50,233 (4935)
Final Rwork	0.1771 (0.2608)
Final Rfree	0.2060 (0.3023)
No. of molecules in the asymmetric unit	2
No. of non-hydrogen atoms	4864
Protein	4563
Ligand	67
Solvent	234
Number of residues	574
Average B-factor values (Å^2^)	50.84
Protein	50.78
Ligand	51.07
Solvent	52.30
RMS bond lengths (Å)	0.0074
RMS bond angles (deg)	0.85
Ramachandran outliers (%)	0.00
Ramachandran favored (%)	98.77
Clash score	7.34
Overall score PDB identifier	1.40 24UU

**Table 2:** Structural alignment RMSD values for CpEndoIV and homologous proteins.

PDB Code	Species	RMSD
8RLY	*Escherichia coli*	1.387
2NQH	*Escherichia coli*	1.392
2NQ9	*Escherichia coli*	1.393
1QTW	*Escherichia coli*	1.406
1QUM	*Escherichia coli*	1.525
2NQJ	*Escherichia coli*	1.534
4K1G	*Escherichia coli*	1.562
4HNO	*Thermotoga maritima*	1.977
2X7V	*Thermotoga maritima*	2.005
2X7W	*Thermotoga maritima*	2.006
3AAM	*Thermus thermophilus HB8*	2.322
3AAL	*Geobacillus kaustophilus*	2.537
8AXY	*Staphylococcus aureus*	2.582
1XP3	*Bacillus anthracis*	2.710
5ZHZ	*Mycobacterium tuberculosis*	3.742

## Data Availability

All generated data and their analyses are shown in the article. The structure factors and coordinates of CpEndoIV have been deposited in the Protein Data Bank under accession number 24UU. Reasonable requests can be directed to the corresponding authors.
